# Foliar P-Fractions Allocation of *Karelinia caspia* and *Tamarix ramosissima* Are Driven by Soil and Groundwater Properties in a Hyper-Arid Desert Ecosystem

**DOI:** 10.3389/fpls.2022.833869

**Published:** 2022-03-31

**Authors:** Yanju Gao, Zhihao Zhang, Bo Zhang, Hui Yin, Xutian Chai, Mengqi Xu, Akash Tariq, Fanjiang Zeng

**Affiliations:** ^1^Xinjiang Key Laboratory of Desert Plant Roots Ecology and Vegetation Restoration, Xinjiang Institute of Ecology and Geography, Chinese Academy of Sciences, Urumqi, China; ^2^State Key Laboratory of Desert and Oasis Ecology, Xinjiang Institute of Ecology and Geography, Chinese Academy of Sciences, Urumqi, China; ^3^Cele National Station of Observation and Research for Desert-Grassland Ecosystems, Cele, China; ^4^University of Chinese Academy of Sciences, Beijing, China

**Keywords:** desert ecosystem, phreatophyte, foliar-P fraction, groundwater, P limitation, soil properties

## Abstract

The allocation patterns of foliar phosphorus (P) fractions across various vegetation types generally reflect the adaptability to P-impoverished environments. However, the allocation of foliar-P fractions within the desert herb *Karelinia caspia* (*K. caspica*) and shrub *Tamarix ramosissima* (*T. ramosissima*) in soils with different environment-P availability and the impact of soil and groundwater properties on foliar-P fractions allocation remain unclear. The foliar-P fractions (metabolites-P, nucleic acid-P, structural-P, and residual-P) of *K. caspica* and *T. ramosissima* and the properties of 0–60 cm deep soil under their canopy and groundwater were determined at four different environment-P sites. Results found that as environment-P availability decreased, both plants allocated the higher proportions of foliar-P to nucleic acid-P than to metabolites-P and structural-P. With the exception of residual-P, foliar-P fractions were markedly higher for *K. caspica* than *T. ramosissima*. Soil Olsen-P, NO_3_^–^-N, soil water content, electrical conductivity (EC), groundwater EC, and total dissolved solids (TDSs) played an important role in allocating foliar P-fractions for both *K. caspica* and *T. ramosissima*. Compared with *K. caspica*, the foliar-P fractions of *T. ramosissima* were more tightly bounded to groundwater than soil properties. Overall, these findings show how desert plants flexibility take advantage of the foliar-P in low environment-P availability and illustrate the foliar-P fractions allocation of desert plants is driven by soil and groundwater properties.

## Introduction

Phosphorus (P) is an essential macronutrient for plants, helping to regulate the metabolism, transportation, and improving the drought resistance of plants (Dobrota, 2004). However, P deficiency in desert ecosystems is a common phenomenon (Hou et al., 2020; Xia et al., 2020). The desert-oasis transition zone on the southern edge of the Taklimakan Desert is a hyper-arid and nutrient-impoverished desert ecosystem (Gao et al., 2022). Vegetation in this region is dominated by sparse perennial herbs and shrubs, which form dominant and co-dominant stands and are important to the security of dunes in the region (Bruelheide et al., 2010; Xue et al., 2019; Zhang et al., 2020). A prior research has found that groundwater is the main source of water and nutrients for deep-rooted desert vegetation in this area (Zeng et al., 2013; Li et al., 2021). However, the annual decline of groundwater table in recent years has brought serious challenges to the vegetation safety in this area (Zeng et al., 2013). Thus, it is crucial to understand the mechanisms that allow flora growing in the juncture zone to accommodate P-impoverished conditions to prevent vegetation degradation and maintain the stability of the juncture zone.

Plants generally respond to P-deficient environments by reducing the leaf P content (Epstein and Bloom, 2005). However, leaf P content is the total of various foliar-P fractions in leaf cells, such as metabolites-P, nucleic acid-P, structural-P, and residual-P (Veneklaas et al., 2012). When faced with low soil-P availability, plants acclimate by shifting the allocation of P between foliar-P fractions (Hidaka and Kitayama, 2011; Yan et al., 2019). Gao et al. (2022) investigated foliar-P fractions in the desert plant, *Alhagi sparsifolia*, along a soil-P availability gradient on the desert-oasis transition zone in Xinjiang, China. In P-impoverished soils, *A. sparsifolia* reduced the metabolites-P content but increased the nucleic acid-P content. In addition, foliar-P fractions allocation may be related to life history because these fractions are functionally associated with growth and reproduction (Hidaka and Kitayama, 2011; Han et al., 2021). For example, Han et al. (2021) found that faster-growing tree species allocate more foliar-P to nucleic acid-P than slow-growing tree species. According to the growth-rate hypothesis (GRH), fast-growing plants need to allocate more foliar-P for protein synthesis and enzyme-related nucleic acid-P (RNA, especially rRNA) to supply photosynthesis and other physiological needs (Elser and Hamilton, 2007; Reef et al., 2010; Hidaka and Kitayama, 2011). A global study on leaf nutrients showed that the leaf P content of faster-growing herbs was significantly higher than in other functional groups (Yuan and Chen, 2009).

The herbaceous perennial, *Karelinia caspia (K. caspica)*, and the shrub, *Tamarix ramosissima* (*T. ramosissima)*, are endemic to the Qira oasis in the Taklimakan Desert (Li et al., 2021; Gao et al., 2022). *K. caspica* is an excellent forage resource that grows relatively fast, and *T. ramosissima* is relatively slow-growing. *T. ramosissima* soil is used by residents to produce the parasitic medicinal plant, *Cistanche tubulosa* (Zhang et al., 2019). Thus, exploring the P utilization of *K. caspica* and *T. ramosissima* is beneficial to ecology and the local economy. Previous studies have shown that *K. caspica* and *T. ramosissima* play a significant role in preventing the land desertification and reducing sand dune movement attributed to “dune” formation (Thompson et al., 2006; Li et al., 2007; Meglioli et al., 2017). A “dune” is formed by the litter of some perennial herbs or shrubs as well as sand movement in desert ecosystems (Throop and Archer, 2008; Sun et al., 2017). In this process of dune formation, plant litter rapidly releases nutrients in response to the special micro-environment under the canopy and forms a “fertile island” (Titus et al., 2002; Thompson et al., 2006; Yao et al., 2019). The creation of fertile islands has been widely reported worldwide and is shown to improve the chemical [e.g., soil organic carbon and total nitrogen (TN)] and biological (e.g., urease and alkaline phosphatase activity) conditions of soil (Meglioli et al., 2017; Yao et al., 2019). The nutrient status of fertile islands formed by diverse vegetation differs based on the litter nutrient concentration, mineralization capacity, and the unique microbial communities of different plants (Innes et al., 2004; Li et al., 2007). Thus, it is worth considering differences in the soil properties of fertile islands formed by *K. caspica* and *T. ramosissima* and how they affect the allocation of foliar-P fractions.

Prior studies have shown that desert ecosystems often have more extensive and deeper roots than vegetation in other ecosystems, likely due to scarce precipitation (Li et al., 2010; Yao et al., 2019). These root characteristics enable desert vegetation to obtain more water and nutrients from both soil and groundwater (Zhang et al., 2018; Li et al., 2021). This feature is attributed to many perennial plants depending on the availability of groundwater in hyper-arid desert ecosystems (Thomas et al., 2006; Bruelheide et al., 2010). Some researchers believe that groundwater is the primary source of water and nutrients for desert vegetation (Gries et al., 2003; Zeng et al., 2013). However, most of the root system of desert vegetation is distributed in the soil, and plant roots only obtain water and nutrients from the ground before they reach the groundwater table. Consequently, the properties of soil and groundwater beneath desert vegetation are equally essential to understand the P nutrition status in desert transition regions.

In this study, the allocation pattern of the relatively fast-growing herb, *K. capsica*, and the relatively slow-growing shrub, *T. ramosissima*, and the impact of soil and groundwater properties on the foliar-P fractions allocation of these plants were assessed in four sites with different environment-P availability (soil Olsen-P and groundwater dissolved P concentration) in the desert-oasis transition zone on the southern edge of the Taklimakan Desert. The study investigated (1) variation of the leaf P and foliar-P fractions contents of *K. caspica* and *T. ramosissima* under different environment-P availability, (2) the allocation pattern of foliar-P fractions in *K. caspica* and *T. ramosissima* at low environment-P availability, and (3) the driving factors of soil and groundwater that influence the allocation of foliar-P fractions in the two desert plants.

## Materials and Methods

### Study Area Description

**FIGURE 1:**
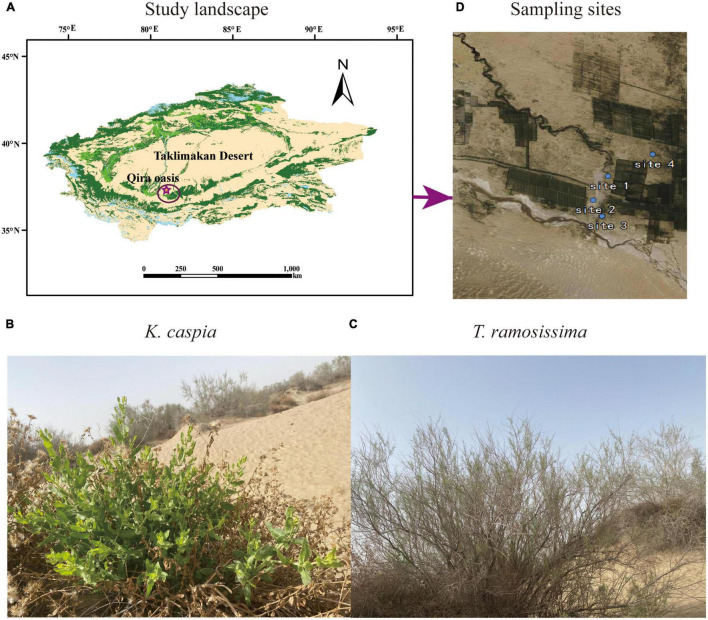
Photos of the study landscape **(A)**, desert species *Karelinia caspia (K. caspica)* **(B)**, *Tamarix ramosissima (T. ramosissima)* **(C)**, and four sampling sites **(D)**.

This study was conducted in the desert-oasis transition zone on the southern edge of the Taklimakan Desert in August 2020 (Figure 1A). In this region, the climate type is warm temperate desert climate, the groundwater depth ranges from 2 to 16 m, the average annual temperature is 15.85°C, the mean annual precipitation during the growing season, June to September is 42.62 mm (hyper-arid), and the maximum evaporation potential is roughly 2,700 mm (Chen et al., 2019). The soil is primarily aeolian sandy soil (Arenosols in the FAO/ISRIC/ISSS, 1998 taxonomy) with high pH and electrical conductivity (EC), low soil organic matter (SOM), soil water content (SWC), and available soil nutrients (Li et al., 2021; Gao et al., 2022). The natural vegetation on this desert-oasis transition zone is dominated by perennial phreatophytes. The perennial shrub, *T. ramosissima*, and herb, *K. capsica*, were selected as study plants for this analysis (Figures 1B,C).

The four study sites had the following longitude, latitude, and groundwater depths: site 1 (N37°01′32′′ E80°42′27′′, 2.5 m), site 2 (N37°00′41′′ E80°42′15′′, 5 m), site 3 (N37°00′34′′ E80°42′28′′, 11 m), and site 4 (N37°00′56′′ E80°43′81′′, 13 m) (Figure 1D). Selection was based on the growth status of the two study plants in each site and the groundwater and soil properties at the depths of 0–60 cm. Soil Olsen-P and groundwater dissolved P concentrations are shown in Tables 1, 2. The distance between two sites was at least 2 km, and the plants selected for each site were at least 500 m far apart.

**TABLE 1:** Soil properties beneath two desert plants at the four study sites.

Soil properties	*K. caspica*				*T. ramosissima*			
	Site 1	Site 2	Site 3	Site 4	Site 1	Site 2	Site 3	Site 4
Olsen-P (mg/kg)	2.03 ± 0.21c	3.75 ± 0.30b	3.86 ± 0.28b	4.69 ± 0.17a	2.17 ± 0.18b***	1.93 ± 0.40bc***	2.42 ± 0.85b***	4.25 ± 0.24a***
NH_4_^+^-N (mg/kg)	1.30 ± 0.35b	1.43 ± 0.25b	1.76 ± 0.14a	1.81 ± 0.19a	1.17 ± 0.33a*	1.15 ± 0.24c***	1.48 ± 0.23b***	1.61 ± 0.26a***
NO_3_^–^-N (mg/kg)	3.24 ± 0.62c	11.53 ± 1.03b	16.19 ± 2.01ab	18.64 ± 4.06a	2.91 ± 0.69d***	10.17 ± 0.74c***	13.14 ± 3.45b***	16.89 ± 4.06a***
SOM (g/kg)	2.18 ± 0.65c	2.10 ± 0.76c	2.36 ± 0.50b	2.64 ± 0.42a	4.10 ± 0.59a***	2.85 ± 0.50b***	2.74 ± 0.31b***	2.76 ± 0.34b***
SWC (%)	2.64 ± 0.59a	0.41 ± 0.03b	0.31 ± 0.06c	0.26 ± 0.10c	7.97 ± 1.04a***	0.30 ± 0.03b***	0.20 ± 0.09c***	0.21 ± 0.15c***
EC (μS/cm)	152.67 ± 11.48d	412.50 ± 62.75c	529.00 ± 14.81b	642.33 ± 32.59a	156.83 ± 6.15d***	420.33 ± 64.07c***	493.50 ± 31.85b***	651.00 ± 30.97a***
pH	8.76 ± 0.10a	8.62 ± 0.17b	8.52 ± 0.10c	8.54 ± 0.07c	8.82 ± 0.07a***	8.69 ± 0.17ab***	8.74 ± 0.08ab***	8.60 ± 0.04b***

*Values are means ± standard deviation (SD, n = 3). Different lower-case letters indicate significant differences among the same plant at four sites (p < 0.05). Asterisks indicate significant differences between the two species in the same soil property by t-test.*

No*p > 0.05; *p < 0.05, ***p < 0.001.

**TABLE 2:** Groundwater properties of the four study sites.

	pH	EC (mS/cm)	Dissolved P (mg/L)	NH_4_^+^-N (mg/L)	NO_3_^–^-N (mg/L)	Total P (mg/L)	Total N (mg/L)	TDSs (g/L)
Site 1	7.72 ± 0.04a	9.20 ± 0.07a	0.03 ± 0.00c	0.75 ± 0.07a	0.36 ± 0.04c	0.04 ± 0.00d	2.50 ± 0.26c	7.67 ± 0.87a
Site 2	7.63 ± 0.02b	2.88 ± 0.75b	0.05 ± 0.01b	0.41 ± 0.02b	0.44 ± 0.03c	0.06 ± 0.01c	1.61 ± 0.07d	3.19 ± 0.93b
Site 3	7.64 ± 0.06b	2.57 ± 0.17c	0.05 ± 0.01b	0.46 ± 0.02b	1.33 ± 0.11b	0.08 ± 0.01b	4.34 ± 0.20b	2.77 ± 0.35c
Site 4	7.68 ± 0.03b	2.62 ± 0.40c	0.13 ± 0.02a	0.69 ± 0.04a	12.32 ± 0.54a	0.19 ± 0.02a	15.36 ± 0.68a	2.37 ± 0.13d

*Values are means ± SD (n = 3). Different lower-case letters indicate significant differences for the four sites (p < 0.05).*

### Plant Samples Collection

At each site, six *T. ramosissima* and *K. caspica* plants with consistent growth status were selected from which soil and plant samples were collected in August 2020, respectively. Approximately 3.0 g young and mature *K. caspica* leaves and 3.0 g mature *T. ramosissima* leaves with no visible damage or discoloration were collected in the natural conditions of each site to ensure enough for 1.0 g dry samples of each leaf type after freeze-drying. Plant samples were kept fresh by immediately placing them in resealable bags, putting them in a car-refrigerator and storing them at –80°C to determine different foliar-P fractions contents. Other leaf samples from the two desert plants were collected from each site to determine the contents of total leaf N and leaf P.

### Soil Samples Collection

After plant sample collection, 0–60 cm deep soil samples were collected along the main root of each plant. The non-decomposed plant litter and sand on the soil surface were removed and a standard vertical soil profile of about 100 cm × 50 cm × 60 cm (length × width × height) was excavated from the center of the main root. The floating soil on the profile was gently swept away, a sample was collected within 2 cm of the main root at every 30 cm soil from bottom to top, and the two soil samples collected on each profile were mixed to obtain a 0–60 cm soil sample. All samples were stored in a car-refrigerator, taken back to the laboratory, and divided into three parts. One part was immediately assessed for soil water content (SWC), the second part was kept at 4°C to measure soil NH_4_^+^-N and NO_3_^–^-N, and the remaining parts were air-dried and used to measured soil Olsen-P, soil organic matter (SOM), EC and pH.

### Groundwater Samples Collection

Groundwater samples were collected from a groundwater observation well at each site. These groundwater observation wells were excavated in 2005. A 1 L plastic bottle with a 2 kg weight at the bottom was extended into the observation well to obtain each groundwater sample and pH, EC, NH_4_^+^-N, NO_3_^–^-N, dissolved P, TN, total P (TP), and total dissolved solids (TDSs) were measured.

### Plant Samples Analysis

Plant leaf P was divided into four fractions: metabolites-P (including inorganic P: Pi), nucleic acid-P, structural-P, and residual-P, and a modified sequential extraction was used for the fractionation procedure (Chapin and Kedrowski, 1983; Hidaka and Kitayama, 2011). In the measurement procedure, a higher weight of each leaf sample was used because of the lower foliar-P content of desert vegetation. Samples were freeze-dried (Alpha 2–4 LD Plus, Christ, Osterode am Harz, Germany), and 1.0 g was weighed into a 10 ml plastic centrifuge tube (tube 1) with 2 ml chloroform: methanol: formic acid (CMF = 12:6:1, v/v/v) and centrifuged for 15 min (5,000 rpm). The supernatant was transferred into another 10 ml plastic centrifuge tube (tube 2), the steps above were repeated, and then, 2.5 ml chloroform: methanol: water (CMW = 1:2:0.8, v/v/v) was added into the initial extraction and the steps above were repeated again. Then, 4 ml water-washed chloroform was added, the tube was centrifuged for 15 min (5,000 rpm), and the lower phase was transferred to 10 ml glass bottle (bottle 1) to determine the structural-P and the upper phase were transferred to another glass bottle (bottle 2). Furthermore, 5 mL methanol (85% v/v) was added into tube 1 and centrifuged for 15 min (5,000 rpm), the supernatant was transferred into bottle 2, and then, 2 ml 5% trichloroacetic acid (TCA) was added and the tube was centrifuged for 10 min (5,000 rpm). These steps were repeated again, the supernatant was transferred into bottle 2 to determine metabolites-P, and another 2 ml 2.5% TCA was added to tube 1. The tube was then placed in a water bath (95°C) for 60 min, cooled, and centrifuged for 10 min (5,000 rpm). These steps were repeated two times before all the supernatant was transferred into a 10 ml glass bottle (bottle 3) to determine nucleic acid-P. Another 5 ml 2.5% TCA was added to tube 1 and transferred to a 10 ml glass bottle (bottle 4) to measure the residual-P. In addition, HNO_3_:H_2_SO_4_ (3:1, v/v) was added into glass bottles 1, 2, 3, and 4 to digest the samples using the molybdenum blue method, and measurement was carried out with a full band spectrophotometer at 620 nm (Scheffer and Pajenkamp, 1952).

Other leaf samples were oven-dried at 75°C for a minimum of 48 h, and then ground and digested in a mixture of concentrated H_2_SO_4_ and H_2_O_2_ until the sample solution was clear. After cooling, the leaf N content was analyzed using an elemental analyzer (2400 II CHNS; Perkin-Elmer, Boston, MA, United States) and the leaf P content was measured using the method developed by Scheffer and Pajenkamp (1952).

### Soil and Groundwater Samples Analysis

Soil Olsen-P (0.5M NaHCO_3_ as extractant), soil TP, groundwater dissolved P, and TP were measured according to the method developed by Olsen and Sommers (1982). Then, NH_4_^+^-N and NO_3_^–^-N concentrations were determined using the AA3 continuous flow analytical system (CFA) after extracting by 2 M KCl. Soil organic matter (SOM) was determined by the wet-oxidation technique (Shaw, 1959). The soil moisture content (SWC) was calculated according to the weight difference between wet soil and dry soil after drying at 105°C for 48 h. The pH and EC were measured at a soil/water ratio of 1:5 (w:v). TN concentration was measured with a full automatic Kjeldahl Nitrogen Analyzer (K1100, Jinan Hanon Instruments Co., Ltd., China). TDSs were gained by evaporating groundwater at 105–120°C.

### Statistical Analysis

Analysis of variance (ANOVA) was performed using SPSS 20.0 software (SPSS Inc., United States). Soil properties, the mass ratios for leaf N to leaf P, and the amount of P in each foliar P-containing fraction were analyzed using one-way ANOVA and *t*-test. Groundwater properties and foliar-P fractions were analyzed using one-way ANOVA. Redundancy analysis was used to analyze the top five environmental factors with the closest correlations between foliar-P fractions and soil and groundwater properties at the four sites. Matrix and circle correlation heatmaps were used to analyze the pairwise and sum correlations among foliar P-fractions and soil and groundwater properties.

## Results

### Soil and Groundwater Properties

**FIGURE 2:**
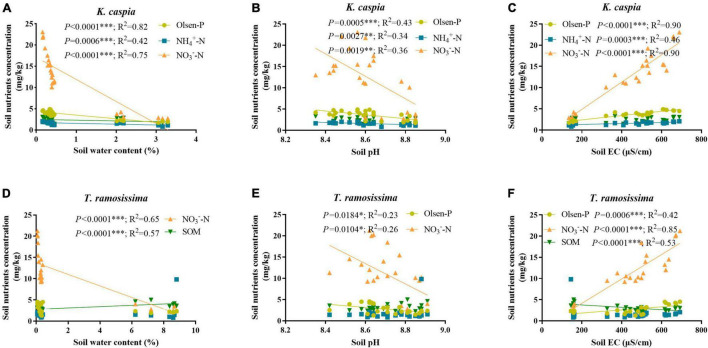
Correlations between the soil basic nutrients and soil water content, pH, and electrical conductivity (EC). The correlation between soil basic nutrients under *K. caspia* and soil water content **(A)**, pH **(B)**, and EC **(C)**, and under *T. ramosissima* and soil water content **(D)**, pH **(E)** and EC **(F)**, respectively. The values of *R*^2^ and *p* are shown only if *p* < 0.05. *p < 0.05, **p < 0.01, ***p < 0.001.

Soil Olsen-P, NH_4_^+^-N, and NO_3_^–^-N concentration increased gradually from site 1 to 4 and was higher in *K. capsica* than *T. ramosissima* samples (Table 1). Compared with *T. ramosissima*, the soil NH_4_^+^-N and NO_3_^–^-N concentration under *K. caspica* were increased by 12.4 and 10.4% at site 4, respectively. The higher nutrient concentration of the soil under *K. caspica* vs. *T. ramosissima* may be attributed to its lower soil pH and EC (Figure 2). SWC and pH were highest at site 1, but EC was the highest at site 4. SOM concentration under *T. ramosissima* decreased from site 1 to 4 and was significantly higher than *K. caspica*. Groundwater dissolved P, NO_3_^–^-N, TP, and TN increased from site 1 to 4, while pH, EC, and TDSs decreased (Table 2).

### Leaf N and P Content

Leaves of *K. caspica* and *T. ramosissima* had similar patterns of leaf N, P, and P in each foliar-P fraction across the four sites (Table 3). With the increasing soil N and P availability (Table 1), compared with the site 1, leaf N, and P of *K. caspica* in site 4 were increased by 31.3 and 18.2%, respectively, and leaf N and P of *T. ramosissima* were increased by 24.8 and 47.1%, respectively. The mean N content in *K. caspica* was decreased by 7.6% relative to *T. ramosissima*, while the mean P content increased by 44.5%. The leaf N:P ratio had a different pattern for the two species across the four sites. The ratio was lower in site 4 than site 1 and higher in *T. ramosissima* than *K. caspica* in every site, indicating that *T. ramosissima* growth was likely limited by P. The leaf N:P_*Fraction*_ ratios of *K. caspica* and *T. ramosissima* for nucleic acid-P were significantly lower in site 1 than site 4, while the ratios for metabolites-P and structural-P were indistinguishable. The ratios of leaf N:P_*Fraction*_ in each P fraction were higher in *T. ramosissima* than *K. caspica* at each site, except that residual-P contributed to the lower leaf N: P ratio.

**TABLE 3:** Mass ratios for total leaf nitrogen (N) to total leaf phosphorus (P) and to P in each foliar P-containing fraction of *Karelinia caspia (K. caspica)* and *Tamarix ramosissima (T. ramosissima)*.

Items	*K. caspica*				*T. ramosissima*			
	Site 1	Site 2	Site 3	Site 4	Site 1	Site 2	Site 3	Site 4
Leaf N	9.97 ± 0.23c	12.22 ± 0.41b	12.61 ± 0.24b	13.09 ± 0.22a	11.63 ± 0.60d***	11.96 ± 0.24c	13.18 ± 0.24b*	14.51 ± 0.78a
Leaf P	0.77 ± 0.09b	0.88 ± 0.07b	1.14 ± 0.02a	0.91 ± 0.12b	0.51 ± 0.05c	0.65 ± 0.02b*	0.65 ± 0.01b***	0.75 ± 0.06a
Leaf N/P	13.11 ± 1.80a	13.98 ± 1.37a	11.06 ± 0.27b	14.58 ± 1.65a	23.38 ± 2.11a*	19.09 ± 1.36c	21.22 ± 1.03b**	19.31 ± 1.48c
Leaf N/metabolites-P	47.77 ± 2.32a	43.07 ± 2.98a	43.07 ± 2.98a	47.55 ± 3.61a	69.75 ± 3.90a**	65.42 ± 1.76a***	52.90 ± 0.64b	62.77 ± 12.38a
Leaf N/nucleic acid-P	32.19 ± 0.78b	37.80 ± 1.42a	38.19 ± 1.91a	36.94 ± 1.07a	45.38 ± 1.83c***	59.01 ± 12.00ab*	50.89 ± 1.30b**	64.79 ± 8.91a**
Leaf N/structural-P	46.49 ± 1.10b	69.45 ± 2.67a	34.00 ± 0.07c	48.22 ± 0.92b	135.55 ± 8.19a***	111.57 ± 5.93b***	96.88 ± 2.70c***	124.91 ± 11.67ab***
Leaf N/residual-P	241.31 ± 21.48b	288.77 ± 20.63a	174.20 ± 0.43d	205.35 ± 4.71c	148.09 ± 16.92ab**	132.05 ± 7.75b***	136.95 ± 5.46b***	157.48 ± 5.98a***

*Values are means ± SD (n = 3). Different lower-case letters indicate significant differences among the same plant at four sites (p < 0.05). Asterisks indicate significant differences between the two species in same soil property by t-test.*

No*p > 0.05; *p < 0.05,**p < 0.01, ***p < 0.001.

### Foliar-P Fractions Content

**FIGURE 3:**
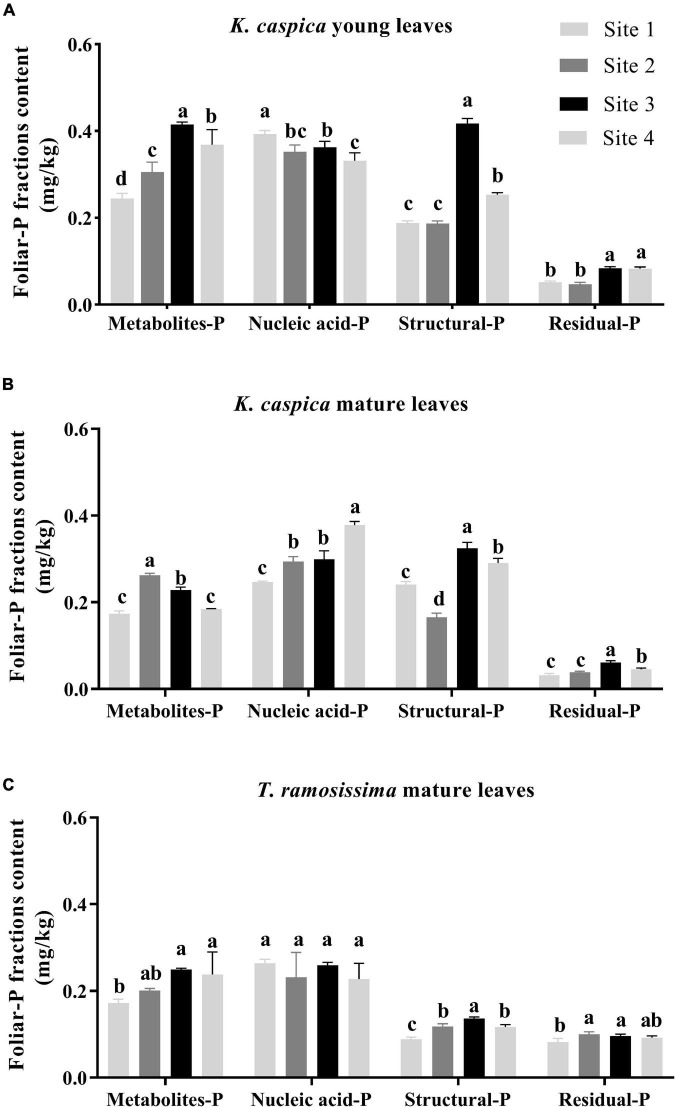
Foliar-P fractions content of two plant species at different environment-P sites. Foliar-P fractions contents of *K. caspia* young leaves **(A)**, *K. caspia* mature leaves **(B)**, and *T. ramosissima* mature leaves **(C)**, respectively. Error bars represent means ± standard deviation (SD, *n* = 3). Different lower-case letters indicate significant differences of the same foliar-P fraction among the sites (*p* < 0.05).

As shown in Figure 3, the foliar-P fractions content of *K. caspica* was distinctly higher than *T. ramosissima*, except for residual-P. Overall, the nucleic acid-P contents of *K. caspica* and *T. ramosissima* were greater than metabolites-P, structural-P, and residual-P, and the young leaves of *K. caspica* were higher than mature leaves and *T. ramosissima*. Meanwhile, the contents of metabolites-P, structural-P, and residual-P in *K. caspica* young leaves and *T. ramosissima* increased as raising the soil available-P concentration. Compared with site 1, the metabolites-P and structural-P contents of *K. caspica* young leaves increased by 50.8 and 34.6% in site 4, while nucleic acid-P reduced by 15.7%. Similarly, the mature leaves of *T. ramosissima* and young leaves of *K. caspica* tended to allocate higher foliar-P to nucleic acid-P than to metabolites-P and structural-P with decreasing soil available-P concentration.

### Allocation Proportions of Foliar-P Fractions

**FIGURE 4:**
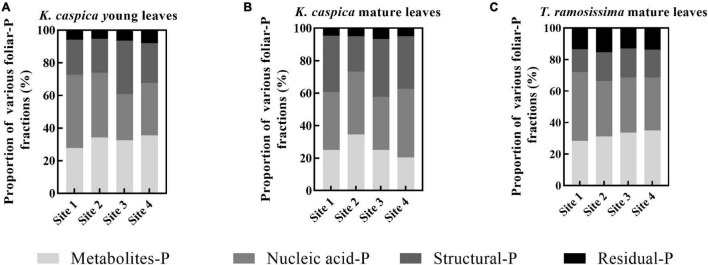
The allocation proportions of foliar-P fractions of two plant species at different environment-P sites. The allocation proportions of foliar-P fractions of *K. caspia* young leaves **(A)**, *K. caspia* mature leaves **(B)**, and *T. ramosissima* mature leaves **(C)**, respectively.

**FIGURE 5:**
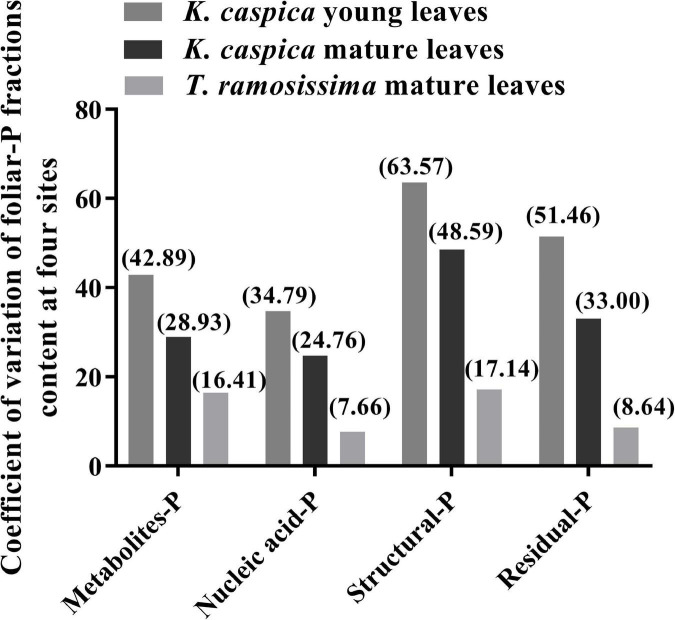
Coefficient of variation of among foliar-P fractions content at the four study sites.

The allocation proportion of nucleic acid-P in *T. ramosissima* mature leaf and *K. caspica* young leaves in site 1 increased by 16 and 18.6% compared with site 4, respectively (Figure 4). In comparison, metabolites-P and structural-P decreased by 33.7, 25.7, 27.8, and 24.2% in site 1. In contrast, the mature leaves of *K. caspica* reduced the allocation proportion of nucleic acid-P as soil available-P concentration decreased. Notably, the allocation proportion of residual-P and nucleic acid-P of *T. ramosissima* were markedly higher than *K. caspica*. The coefficient of variation (*CV*) of foliar-P fractions at the all sites shows that *K. caspica* young leaves > *K. caspica* mature leaves > *T. ramosissima*, structural-P > residual-P > metabolites-P > nucleic acid-P (Figure 5).

### Redundancy Analysis Between the Foliar-P Fractions and Environment Properties

**FIGURE 6:**
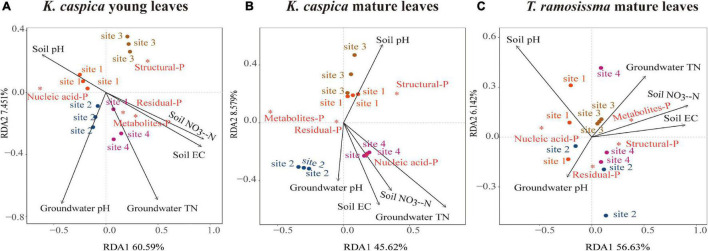
Redundancy analyses of the correlations between foliar-P fractions and soil and groundwater properties. Redundancy analysis on the correlations between soil and groundwater properties and foliar-P fractions of *K. caspia* young leaves **(A)**, *K. caspia* mature leaves **(B)**, and *T. ramosissima* mature leaves **(C)**, respectively. Red asterisk means the foliar-P fractions; black arrow means the soil and groundwater properties. Only the top five environment factors are displayed.

Redundancy analysis was used to analyze the correlations between foliar-P fractions and soil and groundwater properties at four sites (Figure 6). Only the top five environment factors were displayed. The arrow angles between the metabolites-P and residual-P of *K. caspica* young leaves and soil NO_3_^–^-N, EC, and groundwater TN, between nucleic acid-P of *K. caspica* young leaves and soil pH, between nucleic acid-P of *K. caspica* mature leaves and soil NO_3_^–^-N, EC, groundwater TN, and pH were considerably smaller than 90 degrees, indicating positive relationships (Figures 6A,B). However, the metabolites-P and structural-P of *T. ramosissima* correlated positively with soil NO_3_^–^-N, EC, and groundwater TN, and nucleic acid-P correlated positively with soil pH (Figure 6C). In addition, for *K. caspica* young leaves, there were positive correlations between structural-P and site 3, nucleic acid-P and site 1, and metabolites-P and site 4, respectively (Figure 6A). However, for *T. ramosissima* mature leaves, there were strong correlations between structural-P and site 4, nucleic acid-P and site 1, and metabolites-P and site 3, respectively (Figure 6C).

### Correlations Between the Foliar-P Fractions and Environment Properties

**FIGURE 7:**
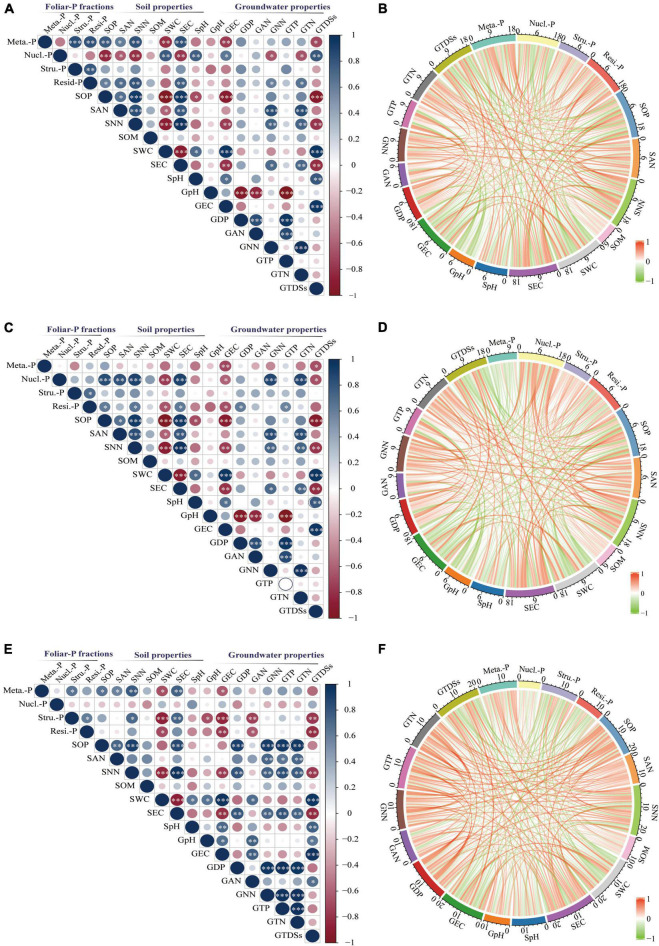
Correlations between foliar-P fractions and soil and groundwater properties. Correlations between soil and groundwater properties and foliar-P fractions of *K. caspia* young leaves **(A,B)**, *K. caspia* mature leaves **(C,D)**, *T. ramosissima* mature leaves **(E,F)**, respectively. Stars represent the level of significance. No*p > 0.05; *p < 0.05, ^**^p < 0.01, ^***^p < 0.001. The sum correlations coefficient between foliar P-fractions and environment factors are expressed by the scale on each arc. Meta.-P: Metabolites-P; Nucl.-P: Nucleic acid-P; Stru.-P: Structural-P; Resi.-P: Residual-P; S: soil; G: groundwater; OP: Olsen-P; DP: dissolved P; AN: NH_4_^+^-N; NN: NO_3_^–^-N; TP: total-P; TN: total-N; SOM: soil organic matter; SWC: soil water content; TDSs: total dissolved solids.

The sum correlations between foliar P-fractions and groundwater and soil properties were analyzed (Figure 7). There was a significant positive relationship between the metabolites-P of *K. caspica* young leaves and soil Olsen-P, NH_4_^+^-N, NO_3_^–^-N, and EC and a negative correlation between the metabolites-P of *K. caspica* young leaves and SWC, groundwater EC, and TDSs (Figure 7A). The pattern was flipped for the nucleic acid-P of *K. caspica* young leaves. However, a significant active correlation between the nucleic acid-P of *K. caspica* mature leaves and soil Olsen-P, NH_4_^+^-N, NO_3_^–^-N, EC, and groundwater NO_3_^–^-N and TN was found (Figure 7C). The metabolites-P and structural-P of *T. ramosissima* were positively correlated with soil Olsen-P, NH_4_^+^-N, NO_3_^–^-N, and EC, but negatively correlated with SWC, groundwater EC and TDSs (Figure 7E).

Of the four foliar-P fractions from the two desert species in this study, the overall relationship of metabolites-P, nucleic acid-P, and residual-P in *K. caspica* young leaves, nucleic acid-P, and residual-P in *K. caspica* mature leaves, and metabolites-P and structural-P in *T. ramosissima* were greater than those of other foliar-P fractions and were more strongly correlated with soil Olsen-P, NO_3_^–^-N, EC, SWC, and groundwater EC and TDSs (Figures 7B,D,F). Furthermore, the overall relationship of foliar-P fractions in *K. caspica* was greater than *T. ramosissima*, while the soil and groundwater properties of *T. ramosissima* were greater than *K. caspica*.

## Discussion

### Variations of Leaf N and P Content

Leaf N and P content are strongly related to leaf photosynthesis and carbon assimilation (Yan et al., 2015). In plant leaves, N and P often represent the best performance of plant adaptability to the environment (Drenovsky et al., 2010). In this study, the leaf P of both *K. caspica* and *T. ramosissima* decreased with decreasing environment-P availability (Table 3). Low environmental-P availability was found to limit P acquisition by plants explaining why they respond to low leaf P content. Indeed, it was previously reported that leaf P was lowest in the most P-impoverished site (Sulpice et al., 2014; Lambers et al., 2015). Additionally, leaf P of the two species in this study was markedly lower than the mean leaf P content in extensive global datasets due to the hyper-arid climate and P-impoverished soil (Wu et al., 2012; Gao et al., 2022). The leaf P content of *K. caspica* was higher than *T. ramosissima* grown in the same site, and *K. caspica* had higher structural-P content, implying that *K. caspica* may have a higher photosynthetic utilization rate (Mo et al., 2019).

Compared with the leaf P content in the two species, the leaf N content of *T. ramosissima* was higher than *K. caspica*. These findings differs from results on *Hakea prostrata* (Proteaceae) which showed that P availability does not affect leaf N content (Prodhan et al., 2019), but correlated leaf N and leaf P content was similar, and enhanced as increasing soil available-P concentration or groundwater available-P concentration (Han et al., 2021). The leaf N content of both species was lower than both those in global soils (Cleveland and Liptzin, 2007; Xu et al., 2013), and lower than results previously reported on *A. sparsifolia* in this area (Zhang et al., 2018; Gao et al., 2022). This is likely explained by the nutrient-impoverished soil in this region, the hyper-arid climate, and the higher soil pH and EC in sandy soil with poor water holding capacity, which reduces the availability, absorption, and transport of nutrients (Figure 2; Gong et al., 2017). In general, the remarkably low leaf N in *K. caspica* and *T. ramosissima* suggests that protein content was low, indicating a low demand for P to adapt to low P availability.

Herbaceous plants have higher leaf P than other functional groups explaining why herbs have low leaf N:P ratios (Yuan and Chen, 2009). This is consistent with results from this study where *T. ramosissima* growth was more likely limited by P while *K. caspica* growth was not. In addition, the higher leaf N content and leaf N:P ratios suggest that more leaf P was allocated to nucleic acid-P (Figure 4; Han et al., 2021). Interestingly, however, the ratios of leaf N:P_*Fraction*_ in each P fraction, with the exception of residual-P, were higher in *T. ramosissima* than in *K. caspica* (Table 3). This suggests that the lower contents of foliar-P fractions with specific functionality may be the main factors limiting P for *T. ramosissima* growing in this area.

### Allocation Patterns of Foliar-P Fractions

Differences in the foliar-P fractions allocation patterns and soil and groundwater properties between the two species reflect distinctions in their nutrient utilization strategies and nutrient source. Aboveground parts of plants, such as herbaceous *K. caspica* die in the winter and resprout again in the spring (Vonlanthen et al., 2011) and have to grow fast to use limited soil, water, and nutrients quickly and efficiently (Verburg et al., 2013), and thus needs the higher P requirement and flexible allocation ability of foliar-P fractions (de Oliveira et al., 2018; Dissanayaka et al., 2018). Leaf P and the *CV* of foliar-P fractions of *K. caspica* were higher than *T. ramosissima* at four sites with different soil-P availability, which just confirmed this viewpoint (Figure 5). Indeed, the contents of foliar structural-P and nucleic acid-P, the balance of membrane lipid metabolism, and the synthesis of enzymes and proteins were higher in *K. caspica* than *T. ramosissima*, to maintain photosynthesis (Figure 3). This strategy may require greater nucleic acid-P investment to support rapid protein synthesis and turnover (Raven, 2012; Jeong et al., 2017). A high protein synthesis capacity may allow plants to acclimate to the variable and changing environments, such as P-impoverished soils or low groundwater dissolved P, and complete their life cycles quickly (Verburg et al., 2013; Mo et al., 2019; Gao et al., 2022). Unlike *K. caspica*, only the leaves of *T. ramosissima* withered and fallen in autumn, a strategy associated with a slower relative growth rate (RGR) (Bruelheide et al., 2010; Vonlanthen et al., 2011). Thus, *T. ramosissima* selection was based on a lower investment in nucleic acid P and the ability to allocate less biomass to deep roots, giving this plant a higher median life span than *K. caspica* (Li et al., 2010; Liu et al., 2016). It was surprising, however, that relatively slow-growing *T. ramosissima* did not have a significantly lower proportion of foliar nucleic acid-P allocation than relatively fast-growing *K. caspica* (Figure 4). The low leaf P content of *T. ramosissima* is most likely explained by extremely low environmental-P in the study region (Gao et al., 2022). To ensure its normal growth and development, *T. ramosissima* allocated more foliar-P proportion to nucleic acid-P to increase hydrolyses and protein synthesis. This is confirmed by the high leaf N: P ratios of *T. ramosissima* (Table 3).

The metabolites-P and structural-P content were significantly greater for *K. caspica* than *T. ramosissima*, suggesting that *K. caspica* has a higher photosynthetic rate and metabolic activity (Figure 3; Mo et al., 2019). Indeed, the content of structural-P is greater in herbs than in other functional groups, and this is attributed to the higher need of herbs to maintain a balance between membrane lipid metabolism and enzyme and protein synthesis (Jeong et al., 2017; Dissanayaka et al., 2018). This may be explained by the larger, fresher, and tenderer leaf characteristics of *K. caspica* (Figure 1B). However, *T. ramosissima* had a higher residual-P proportion of total P than *K. caspica*, and the proportion was lower when grown in the lower soil-P availability of site 1 than sites 2, 3, and 4 (Figure 4). The foliar residual-P differences between the two species may be attributed to a need for more metabolites-P and nucleic acid-P to participate in metabolic activities and the higher photosynthetic demand of fast-growing herbs than slow-growing shrubs (Mo et al., 2019; Gao et al., 2022). Although residual-P concentration is relatively constant in the same species, phosphatases may dephosphorylate phosphorylated proteins in extremely P-impoverished environments (Schlüter et al., 2013), explaining why the proportion of residual-P was lowest in the site with the least environmental-P (site 1).

### Effect of Different Soil and Groundwater Properties on Foliar-P Fractions Allocation Patterns

Nucleic acid-P was negatively correlated with soil-P availability and groundwater dissolved P concentration in our study, while metabolites-P was positively correlated (Figures 6, 7). This suggests that low environment-P allows plants to distribute more foliar-P fractions to nucleic acid-P to promote hydrolase and protein synthesis, and then provides more P to metabolites-P fraction (Raven, 2012; Jeong et al., 2017; Dissanayaka et al., 2018).

Foliar-P fractions’ allocation patterns in the two desert plants were tightly related to soil Olsen-P, NO_3_^–^-N, SWC, EC, and groundwater EC and TDSs in this area (Figures 6, 7). This indicated that soil and groundwater properties were both crucial drivers of foliar-P fractions allocation by desert plants in a hyper-arid desert ecosystem. This is corroborated with the earlier findings as reported by Gao et al. (2022). However, compared with *K. caspica*, the foliar-P fractions of *T. ramosissima* were more closely related to groundwater than soil properties (Figure 7). This may be explained by the root characteristics of shrubs and herbs. Liu et al. (2016) monitored the fine roots of some perennial herbs, shrubs, and tree species in the Taklimakan desert and showed that *K. caspica* had a primary anatomical structure and higher N content, greater respiration rate, and a shorter life span than *T. ramosissima*. The fine roots of *T. ramosissima* showed thicker diameters, no cortex or a collapsed cortex, and distinct secondary growth. These findings suggest that *K. caspica* can absorb more nutrients and water from the soil than *T. ramosissima*. Since fine root structure generally determines nutrient and water-absorbing ability (McCormack et al., 2012, 2013, 2015), the stronger vitality of fine roots is conducive to nutrient and water absorption (Liu et al., 2016).

**FIGURE 8:**
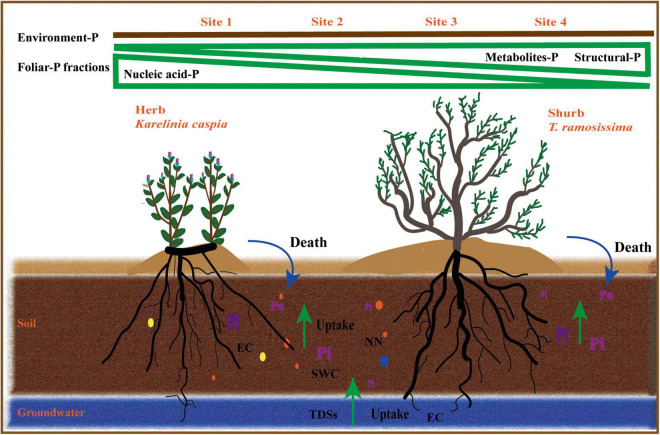
Diagrams summarizing foliar-P fractions allocation of two plant species at different environment-P levels.

Prior studies found that *K. caspica* has a deeper root than *T. ramosissima*, as well as clonal reproduction (Li et al., 2010; Vonlanthen et al., 2011), indicating that *K. caspica* receives more nutrients and water from both soil and groundwater. Similarly, the results presented here showed that while foliar-P fractions allocation of *K. caspica* was influenced by many soil and groundwater properties, the foliar-P fractions allocation of *T. ramosissima* was more closely related to groundwater properties. Overall, the two species have a similar pattern of foliar-P fractions allocation but their responses to soil and groundwater properties are completely different. These differences may be associated with growth strategies designed for the particular ecological niches in which they are found (Figure 8).

## Conclusion

This study provides strong evidence that not all desert vegetation growing in P-impoverished soils are limited by environment-P; *T. ramosissima* was limited, while *K. caspica* was not. This may be because *K. caspica* had higher soil-P levels than *T. ramosissima*. The high content and allocation proportion of nucleic acid-P and the low content and allocation proportions of metabolites-P and structural-P suggested that the allocation patterns of foliar-P fractions by the herb and shrub were similar in the P-impoverished study sites. Groundwater properties were another driver of foliar-P fractions allocation in this study. These results supply an essential theoretical basis for understanding the differences in foliar-P fractions allocation between fast-growing desert herbs and slow-growing desert shrubs in regions with different environmental-P availability. However, understanding of why residual-P was significantly higher in the shrub than the herb, what role residual-P plays in foliar-P fractions allocation, and the correlation between shrub and groundwater nutrients requires further exploration.

## Data Availability Statement

The original contributions presented in the study are included in the article/supplementary material, further inquiries can be directed to the corresponding author/s.

## Author Contributions

YG and FZ planned and designed the research. YG, MX, and XC conducted the fieldwork. YG, ZZ, HY, and BZ analyzed the data. AT and FZ revised the manuscript. All authors contributed to the article and approved the submitted version.

## Conflict of Interest

The authors declare that the research was conducted in the absence of any commercial or financial relationships that could be construed as a potential conflict of interest.

## Publisher’s Note

All claims expressed in this article are solely those of the authors and do not necessarily represent those of their affiliated organizations, or those of the publisher, the editors and the reviewers. Any product that may be evaluated in this article, or claim that may be made by its manufacturer, is not guaranteed or endorsed by the publisher.
